# The influence of marital status on the survival of patients with esophageal cancer: a population-based, propensity-matched study

**DOI:** 10.18632/oncotarget.19446

**Published:** 2017-07-22

**Authors:** Qing-Wei Zhang, Xiao-Lu Lin, Chi-Hao Zhang, Chen-Yue Tang, Xin-Tian Zhang, La-Mei Teng, Zhi-Zheng Ge, Xiao-Bo Li

**Affiliations:** ^1^ Division of Gastroenterology and Hepatology, Key Laboratory of Gastroenterology and Hepatology, Ministry of Health, Renji Hospital, School of Medicine, Shanghai Jiao Tong University, Shanghai Institute of Digestive Disease, Shanghai 200001, China; ^2^ Department of General Surgery, Shanghai Ninth People’s Hospital, School of Medicine, Shanghai Jiao Tong University, Baoshan 201999, Shanghai, China; ^3^ Department of Gastroenterology, Ruijin Hospital, Shanghai Jiao Tong University School of Medicine, Shanghai 200000, China; ^4^ Division of Gastroenterology and Hepatology, Liqun Clinical Medicine College, The Second Military Medical University, Liqun Hospital, Shanghai 200001, China; ^5^ Department of Digestive Endoscopy, Provincial Clinic Medical College, Fujian Medical University, Fujian Provincial Hospital, Fuzhou 350001, China

**Keywords:** esophageal cancer, marital status, SEER, OS, CSS

## Abstract

**Background and aims:**

Multiple studies have shown that marital status is associated with the survival of various types of cancer patients. However, there has not been adequate evidence of the association between marital status and the survival of patients with esophageal cancer (EC). We aimed to investigate the effect of marital status on survival of EC patients.

**Methods:**

We identified 15,598 EC patients from the Surveillance, Epidemiology, and End Results (SEER) database. Meanwhile, propensity scores for marital status, which were calculated for each patient using a nonparsimonious multivariable logistic regression model, were used to match 6,319 unmarried patients with 9,279 married patients. We performed Kaplan–Meier analysis and multivariate Cox regression to analyze the association between marital status and the overall survival (OS) and EC cause-specific survival (CSS) of EC patients before matching and after matching.

**Results:**

We matched 2,986 unmarried patients with 2,986 married patients. Unmarried patients had poorer OS than married patients before matching (hazard ratio [HR]: 1.22; 95% confidence interval [CI]: 1.18–1.27; P < 0.0001) and after matching (HR: 1.20; 95% CI: 1.13–1.27; P < 0.0001) and poorer CSS than married patients before matching (HR: 1.21; 95% CI: 1.16–1.26; P < 0.0001) and after matching (HR: 1.17; 95% CI: 1.10–1.24; P < 0.0001). Further analysis showed that among different unmarried patients, widowed patients had the poorest OS (HR: 1.46; 95% CI: 1.38–1.55; P < 0.0001) and CSS (HR: 1.43; 95% CI: 1.34–1.52; P < 0.0001) compared with married patients.

**Conclusions:**

Unmarried EC patients had poorer survival rates than married EC patients. Meanwhile, widowed patients with EC had the highest risk of death compared with single, married, and divorced patients.

## INTRODUCTION

Esophageal cancer (EC) is one of ten leading causes of cancer-related deaths, with 16,940 new cases and 15,690 deaths in the US [1]. Although the management and treatment of EC has been improved in recent years, the survival of patients with EC is still poor, with a five-year survival rate of 22% [2]. Several clinical characteristics have proved to be associated with poor patient survival, including tumor grade, tumor stage at diagnosis, and whether surgery or adjuvant therapy was performed. Socioeconomic factors are also likely to contribute to the survival of patients with EC, which is an association that has been demonstrated in other cancers [3–7].

One socioeconomic factor, marital status, contributes to better health with various diseases, including malignancies [8]. Previous studies have demonstrated that marital status played an important role in patients’ survival of various cancers, such as liver, gastric, colorectal, and pancreatic cancers [4–7]. However, conclusions about the contribution of marital status to patients’ survival have contradicted each other [3, 9, 10]. One population-based nationwide Swedish cohort study that included patients with gastric cancer or EC found that unmarried patients had poorer survival rates than married patients [9]. On the other hand, a prospective population-based cohort that included postsurgery EC patients from all Swedish hospitals investigated the role of marital status in EC survival but found that unmarried patients did not have poorer survival rates than married patients [10]. In a large population-based study that used data from the Surveillance, Epidemiology, and End Results (SEER) database, the authors used the Cox proportional hazards multivariable regression while adjusting for age, sex, race, income, residence, nodal stage, tumor stage, educational level, metastatic status, and use of definitive therapy to analyze the role of marital status in the ten leading cancers that cause cancer-related deaths, including EC, concluding that unmarried patients had a higher risk death from these cancers [3].

However, marital statuses have changed in recent years [11]. In addition, delayed diagnosis, rejection of therapy, and a lack of social support could contribute to poor prognosis of cancers, which could all also be influenced by marital status [12, 13]. Therefore, the potential association between marital status and EC survival is still unclear and should be analyzed.

We conducted a study that involved a large population consisting of patients diagnosed between 2004 and 2012 and that included information about tumor grade, tumor histology, location of the EC, and more detailed therapy information. In this study, which was based on Aizer’s study [3], we further explored whether different marital statuses could affect the survival of EC patients. This study employed propensity score matching (PSM), which is an effective tool to reduce selection and residual biases. Furthermore, we used Cox proportional hazards multivariable regression to reanalyze the role of marital status in the survival of EC patients in the matched cohort using PSM with a balance of studied exposure variables between married and unmarried patients.

## RESULTS

### Patient characteristics

In total, 15,598 patients with EC who met our inclusion criteria, including 9,279 (59.49%) married patients and 6,319 (40.51%) unmarried patients, were identified in the SEER database. Of the unmarried patients, 1,971 (12.62%) were divorced, 2,592 (16.62%) were single, and 1,756 (11.26%) were widowed. Table 1 describes the clinicopathological characteristics of the patients with different marital statuses.

**Table 1:** Characteristics of patients with esophageal carcinomas in SEER database

Characteristics	Total	Married	Unmarried	Divorced	Single	Widowed
	15598 (100%)	9279 (59.49%)	6319 (40.51%)	1971 (12.64%)	2592 (16.62%)	1756 (11.26%)
**Sex**						
Male	12581 (80.66%)	8093 (87.22%)	4488 (71.02%)	1555 (78.89%)	2083 (80.36%)	850 (48.41%)
Female	3017 (19.34%)	1186 (12.78%)	1831 (28.98%)	416 (21.11%)	509 (19.64%)	906 (51.59%)
**Race**						
White	13241 (84.89%)	8297 (89.42%)	4944 (78.24%)	1629 (82.65%)	1879 (72.49%)	1436 (81.78%)
Black	1583 (10.15%)	480 (5.17%)	1103 (17.46%)	273 (13.85%)	609 (23.50%)	221 (12.59%)
Other race	774 (4.96%)	502 (5.41%)	272 (4.30%)	69 (3.50%)	104 (4.01%)	99 (5.64%)
**Age**						
<40	216 (1.38%)	103 (1.11%)	113 (1.79%)	16 (0.81%)	95 (3.67%)	2 (0.11%)
41-55	2985 (19.14%)	1604 (17.29%)	1381 (21.85%)	466 (23.64%)	863 (33.29%)	52 (2.96%)
56-70	7233 (46.37%)	4565 (49.20%)	2668 (42.22%)	1062 (53.88%)	1175 (45.33%)	431 (24.54%)
71-85	4416 (28.31%)	2687 (28.96%)	1729 (27.36%)	399 (20.24%)	396 (15.28%)	934 (53.19%)
>85	748 (4.80%)	320 (3.45%)	428 (6.77%)	28 (1.42%)	63 (2.43%)	337 (19.19%)
**Histology**						
ESCC	4725 (30.29%)	2231 (24.04%)	2494 (39.47%)	696 (35.31%)	1071 (41.32%)	727 (41.40%)
EAC	9530 (61.10%)	6193 (66.74%)	3337 (52.81%)	1119 (56.77%)	1348 (52.00%)	870 (49.54%)
Others	1343 (8.61%)	855 (9.21%)	488 (7.72%)	156 (7.91%)	173 (6.67%)	159 (9.05%)
**Grade**						
Well differentiated	844 (5.41%)	507 (5.46%)	337 (5.33%)	100 (5.07%)	136 (5.25%)	101 (5.75%)
Moderately differentiated	6276 (40.24%)	3592 (38.71%)	2684 (42.48%)	834 (42.31%)	1120 (43.21%)	730 (41.57%)
Poorly differentiated	8172 (52.39%)	5002 (53.91%)	3170 (50.17%)	1009 (51.19%)	1290 (49.77%)	871 (49.60%)
Undifferentiated	306 (1.96%)	178 (1.92%)	128 (2.03%)	28 (1.42%)	46 (1.77%)	54 (30.75%)
**Location**						
Upper third of esophagus	1053 (6.75%)	481 (5.18%)	572 (9.05%)	167 (8.47%)	245 (9.45%)	160 (9.11%)
Middle third of esophagus	3186 (20.43%)	1595 (17.19%)	1591 (25.18%)	459 (23.29%)	655 (25.27%)	477 (27.16%)
Lower third of esophagus	11359 (72.82%)	7203 (77.63%)	4156 (65.77%)	1345 (68.24%)	1692 (65.28%)	1119 (63.72%)
**TNM Stage**						
Stage I	2548 (16.34%)	1495 (16.11%)	1053 (16.67%)	266 (13.50%)	385 (14.85%)	402 (22.89%)
Stage II	3317 (21.27%)	1949 (21.00%)	1368 (21.65%)	428 (21.71%)	524 (20.22%)	416 (23.69%)
Stage III	3733 (23.93%)	2299 (24.78%)	1434 (22.69%)	487 (24.71%)	595 (22.96%)	352 (20.04%)
Stage IV	6000 (38.47%)	3536 (38.11%)	2464 (38.99%)	790 (40.08%)	1088 (41.98%)	586 (33.37%)
**Therapy**						
No surgery or radiotherapy	10247 (65.69%)	5608 (60.44%)	4639 (73.41%)	1371 (69.56%)	1854 (71.53%)	1414 (80.52%)
Only surgery	2107 (13.51%)	1393 (15.01%)	714 (11.30%)	224 (11.36%)	319 (12.31%)	171 (9.74%)
Only radiotherapy	359 (2.30%)	224 (2.41%)	135 (2.14%)	45 (2.28%)	62 (2.39%)	28 (1.59%)
Surgery and radiotherapy	2885 (18.50%)	2054 (22.14%)	831 (13.15%)	331 (16.79%)	357 (13.77%)	143 (8.14%)

SEER=surveillance, epidemiology and end results; ESCC=esophageal squamous cell carcinoma; EAC=esophageal adenocarcinoma; TNM= tumor, node and metastasis.

As shown in Table 1, compared with the male patients, more female patients tended to be unmarried. Among the widowed patients, 906 (51.59%) were female, compared with proportion of females in patients with other marital statuses. With respect to the patients’ race, Black patients were more likely to be unmarried than White patients and patients of other races. Compared with married patients, unmarried patients were more likely to receive no surgery or radiotherapy, to have esophageal adenocarcinoma (EAC), and to have cancers located in the middle third of the esophagus. However, there seemed to be little difference in the patients’ TNM stage, which had a standard difference (SD) of 0.049, and tumor grade (SD: 0.079). The detailed clinicopathological characteristics of the patients with different marital statuses are shown in Table 1.

### The effects of marital status on overall survival and cause-specific survival in the unmatched 15,598-patient cohort with esophageal cancer

**Figure 1:**
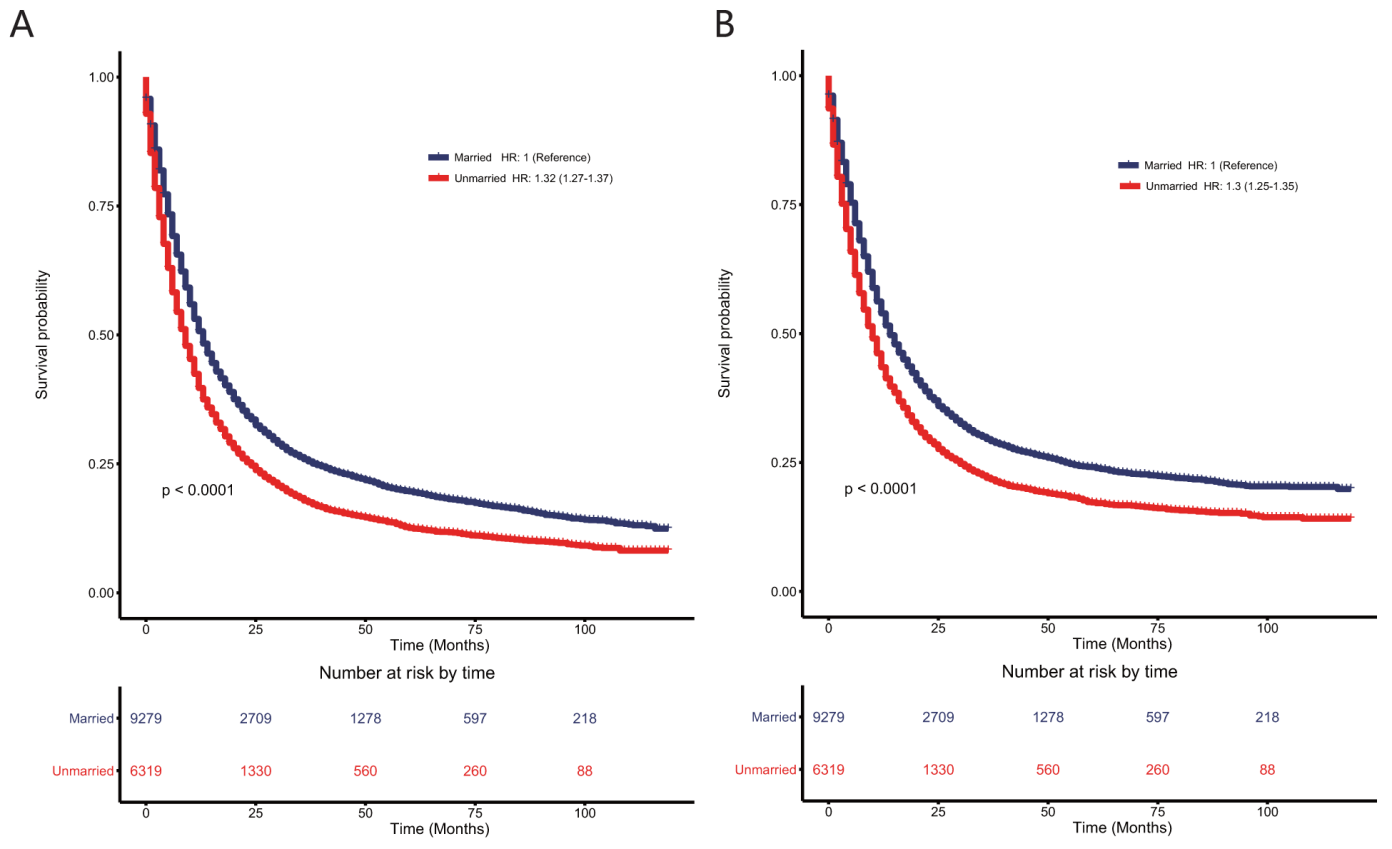
Kaplan–Meier survival plots of the 15,558 patients with esophageal cancers according to marital status **(A)** overall survival; **(B)** esophageal cancer cause-specific survival.

Kaplan–Meier curves were used to evaluate the overall survival (OS) rate of EC patients (see Figure 1A). As shown in Figure 1A, unmarried patients had poorer prognoses (hazard ratio [HR]: 1.32; 95% confidence interval [CI]: 1.27–1.37; P < 0.0001) than married patients according to the Cox proportional hazards univariate regression model. After controlling the patients’ baseline characteristics, including age, sex, race, therapy, TNM stage, tumor grade, tumor location, and tumor histology, unmarried patients still had poorer prognoses (HR: 1.22; 95% CI: 1.18–1.27; P < 0.0001) than married patients.

The cause-specific survival rates (CSS) of EC patients were also plotted using Kaplan–Meier curves. As shown in Figure 1B, compared with being married, being unmarried contributed to poor prognoses (HR: 1.30; 95% CI: 1.25–1.35; P < 0.0001) according to the Cox proportional hazards univariate regression model and even when controlling confounding factors using the Cox proportional hazards multivariate regression model (HR: 1.21; 95% CI: 1.16–1.26; P < 0.0001).

In addition, age, sex, race, tumor grade, tumor stage, tumor location, and type of therapy were validated as independent prognosis factors for OS and CSS in the multivariate Cox analyses. The detailed description of each prognosis factor is shown in Table 2.

**Table 2:** Univariate and multivariate Cox regression analysis for evaluating the influence of marital status on OS and esophageal cancer CSS in 15598 unmatched cohort with esophageal cancer in SEER database

Variable	OS				CSS			
	Univariate analysis		Multivariate analysis		Univariate analysis		Multivariate analysis	
	HR (95% CI)	P	HR (95% CI)	P	HR (95% CI)	P	HR (95% CI)	P
**Marriage**								
Married	Reference		Reference		Reference		Reference	
Unmarried	1.28 (1.24-1.33)	<0.001	1.22 (1.18-1.27)	<0.001	1.26 (1.21-1.31)	<0.001	1.21 (1.16-1.26)	<0.001
**Sex**								
Male	Reference		Reference		Reference		Reference	
Female	0.99 (0.95-1.04)	0.854	0.86 (0.82-0.90)	<0.001	1.00 (0.95-1.05)	0.950	0.88 (0.84-0.93)	<0.001
**Race**								
White	Reference		Reference		Reference		Reference	
Black	1.32 (1.24-1.40)	<0.001	1.16 (1.09-1.24)	<0.001	1.30 (1.22-1.38)	<0.001	1.15 (1.07-1.23)	<0.001
Other race	1.01 (0.93-1.10)	0.763	0.94 (0.86-1.02)	0.154	1.01 (0.92-1.11)	0.823	0.94 (0.86-1.03)	0.218
**Age**								
(<40) vs (41-55) vs (56-70) vs (71-85) vs (>85)	1.21 (1.18-1.24)	<0.001	1.19 (1.16-1.22)	<0.001	1.16 (1.14-1.19)	<0.001	1.16 (1.14-1.19)	<0.001
**Histology**								
ESCC	Reference		Reference		Reference		Reference	
EAC	0.84 (0.81-0.88)	<0.001	0.99 (0.93-1.05)	0.649	0.86 (0.82-0.9)	<0.001	0.86 (0.82-0.9)	<0.001
Others	1.14 (1.06-1.22)	<0.001	1.14 (1.05-1.24)	0.001	1.17 (1.10-1.27)	<0.001	1.17 (1.10-1.27)	<0.001
**Grade**								
I vs II vs III vs IV	1.30 (1.27-1.34)	<0.001	1.17 (1.14-1.21)	<0.001	1.36 (1.31-1.40)	<0.001	1.21 (1.17-1.25)	<0.001
**Location**								
Upper third of esophagus	Reference		Reference		Reference		Reference	
Middle third of esophagus	1.03 (0.95-1.11)	0.517	1.15 (1.07-1.25)	<0.001	1.03 (0.95-1.13)	0.373	1.17 (1.07-1.27)	0.001
Lower third of esophagus	0.90 (0.84-0.97)	0.007	1.13 (1.04-1.23)	0.003	0.93 (0.86-1.01)	0.080	1.14 (1.04-1.24)	0.004
**TNM Stage**								
I vs II vs III vs IV	1.55 (1.52-1.58)	<0.001	1.38 (1.36-1.41)	<0.001	1.65 (1.62-1.69)	<0.001	1.46 (1.43-1.49)	<0.001
**Therapy**								
Surgery and radiotherapy	Reference		Reference		Reference		Reference	
Only surgery	0.80 (0.74-0.86)	<0.001	1.01 (0.94-1.03)	0.445	0.74 (0.68-0.81)	<0.001	0.99 (0.91-1.08)	0.817
Only radiotherapy	2.08 (1.84-2.35)	<0.001	1.63 (1.44-1.85)	<0.001	2.22 (1.95-2.53)	<0.001	1.68 (1.48-1.92)	<0.001
No surgery or radiotherapy	2.94 (2.79-3.09)	<0.001	2.44 (2.31-2.57)	<0.001	3.04 (2.87-3.21)	<0.001	2.49 (2.35-2.64)	<0.001

SEER=surveillance, epidemiology and end results; OS=overall survival; CSS=cause-specific survival; ESCC=esophageal squamous cell carcinoma; EAC=esophageal adenocarcinoma; TNM= tumor, node and metastasis.

### The effects of different unmarried statuses on overall survival and cause-specific survival in the unmatched 15,598-patient cohort with esophageal cancer

**Figure 2:**
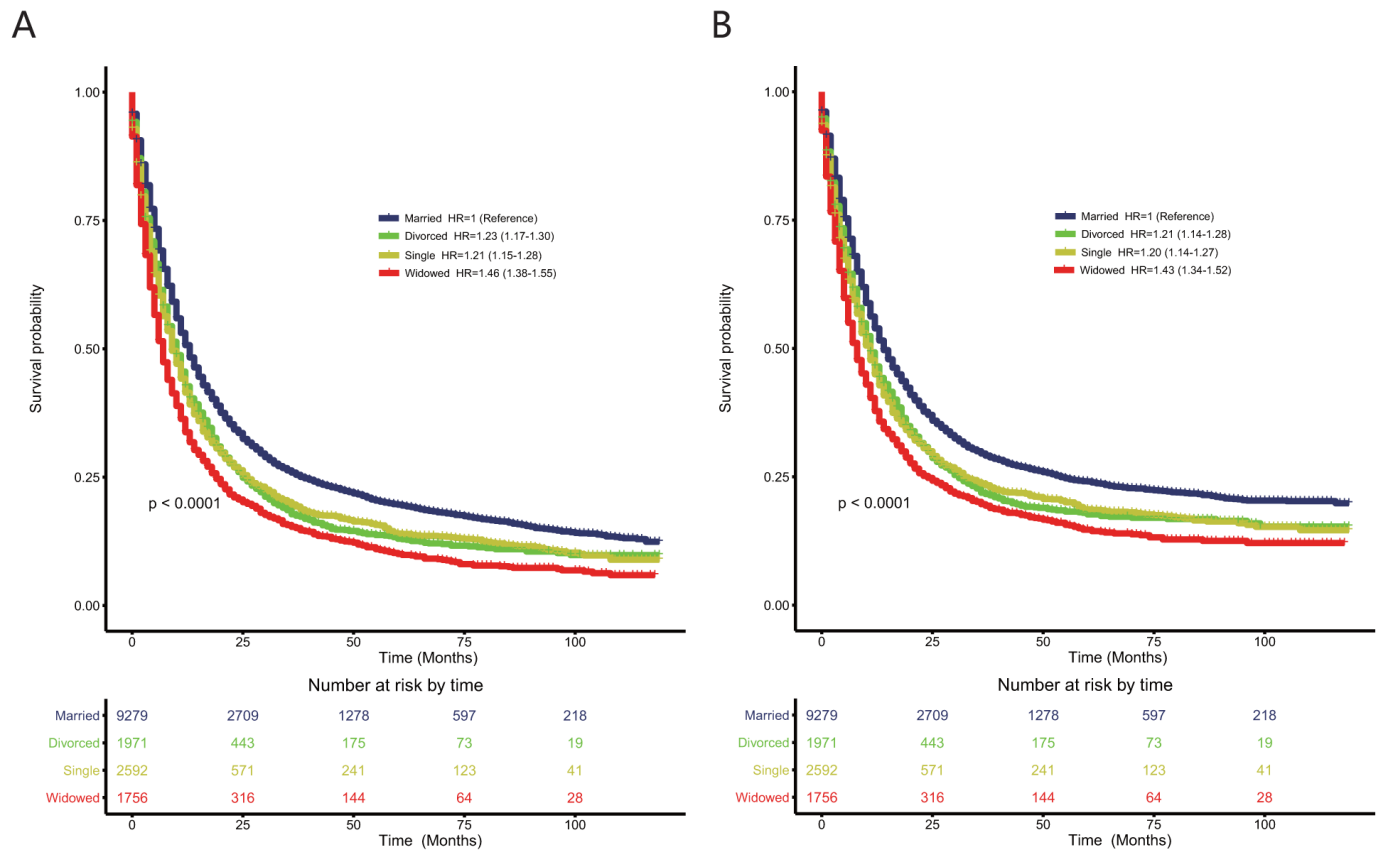
Kaplan–Meier survival plots of the 15,558 patients with esophageal cancers among single, married, widowed, and divorced patients **(A)** overall survival; **(B)** esophageal cancer cause-specific survival.

To explore whether different unmarried statuses contributed to poorer prognoses than being married, we divided unmarried patients into three subgroups: single, widowed, and divorced. As shown in Figure 2A, compared with married patients, divorced patients (HR: 1.23; 95% CI: 1.17–1.30; P < 0.0001) and single patients (HR: 1.21; 95% CI: 1.15–1.28; P < 0.0001) had similarly poor prognoses according to the Cox proportional hazards univariate regression model. Widowed patients had the poorest OS (HR: 1.46; 95% CI: 1.38–1.55; P < 0.0001). After controlling all baseline characteristics using the Cox proportional hazards multivariate regression model, divorced patients (HR: 1.20; 95% CI: 1.14–1.27; P < 0.0001), single patients (HR: 1.22; 95% CI: 1.15–1.28; P < 0.0001), and widowed patients (HR: 1.25; 95% CI: 1.17–1.33; P < 0.0001) all had poorer OS than married patients.

As shown in Figure 2B, compared with married patients, divorced patients (HR: 1.21; 95% CI: 1.14–1.28; P < 0.0001) and single patients (HR: 1.20; 95% CI: 1.14–1.27; P < 0.0001) had poorer CSS using the Cox proportional hazards univariate regression model. Widowed patients had the poorest CSS (HR: 1.43; 95% CI: 1.34–1.52; P < 0.0001). After controlling all baseline characteristics, divorced patients (HR: 1.18; 95% CI: 1.11–1.25; P < 0.0001), single patients (HR: 1.20; 95% CI: 1.13–1.27; P < 0.0001), and widowed patients (HR: 1.25; 95% CI: 1.17-1.34; P < 0.0001) all had poorer OS than married patients.

### Subgroup analysis of the effects of different marital statuses on overall survival and cause-specific survival in the unmatched 15,598-patient cohort with esophageal cancer according to tumor stage

Since widowed patients appeared to have more early stage EC, we analyzed whether unmarried status or single, divorced, or widowed status contributed to the poor survival rates in the subgroups of EC patients with different tumor stages according to American Joint Committee on Cancer (AJCC) staging (sixth edition). Using univariate and multivariate Cox regression analysis, some interesting issues were found. First, unmarried status contributed to poor OS and CSS in each group of patients with different AJCC stage tumors in the univariate and multivariate Cox model (P < 0.001). Second, when the unmarried group was divided into the single, widowed, and divorced groups, the widowed patients had the poorest OS and CSS of the patients with stage I, II, or III EC in the univariate and multivariate Cox model compared with the married, single, and divorced groups. However, in patients with stage IV EC, single patients had the poorest OS and CSS. Detailed information about the OS and CSS of the married patients is shown in Tables 3 and 4. In addition, the subgroup analysis based on different baseline characteristics is shown in Supplementary Tables 1 and 2.

**Table 3:** Univariate and multivariate Cox regression analysis of unmarried status compared with married status on OS and esophageal cancer CSS based on different 6^th^ AJCC stage in 15598 unmatched cohort with esophageal cancer

Variable	OS				CSS			
	Univariate analysis		Multivariate analysis		Univariate analysis		Multivariate analysis	
	HR (95% CI)	P	HR (95% CI)	P	HR (95% CI)	P	HR (95% CI)	P
**TNM Stage**								
**Stage I**								
Married	Reference		Reference		Reference		Reference	
Unmarried	1.59 (1.44-1.76)	<0.001	1.27 (1.14-1.42)	<0.001	1.70 (1.52-1.92)	<0.001	1.30 (1.15-1.47)	<0.001
**Stage II**								
Married	Reference		Reference		Reference		Reference	
Unmarried	1.27 (1.17-1.38)	<0.001	1.19 (1.09-1.30)	<0.001	1.25 (1.14-1.37)	<0.001	1.17 (1.06-1.29)	0.002
**Stage III**								
Married	Reference		Reference		Reference		Reference	
Unmarried	1.36 (1.26-1.46)	<0.001	1.28 (1.18-1.38)	<0.001	1.33 (1.22-1.43)	<0.001	1.25 (1.15-1.36)	<0.001
**Stage IV**								
Married	Reference		Reference		Reference		Reference	
Unmarried	1.22 (1.15-1.29)	<0.001	1.20 (1.13-1.27)	<0.001	1.20 (1.13-1.27)	<0.001	1.18 (1.11-1.25)	<0.001

AJCC= American Joint Committee on Cancer; OS=overall survival; CSS=cause-specific survival; TNM= tumor, node and metastasis.

**Table 4:** Univariate and multivariate Cox regression analysis of divorced, single and widowed status compared with married status on OS and esophageal cancer CSS based on different 6^th^AJCC stage in 15598 unmatched cohort with esophageal cancer

Variable	OS				CSS			
	Univariate analysis		Multivariate analysis		Univariate analysis		Multivariate analysis	
	HR (95% CI)	P	HR (95% CI)	P	HR (95% CI)	P	HR (95% CI)	P
**TNM Stage**								
**Stage I**								
Married	Reference		Reference		Reference		Reference	
Divorced	1.41 (1.19-1.66)	<0.001	1.37 (1.16-1.63)	<0.001	1.47 (1.22-1.78)	<0.001	1.37 (1.13-1.67)	<0.001
Single	1.21 (1.05-1.41)	0.011	1.14 (0.98-1.32)	0.100	1.24 (1.05-1.48)	0.013	1.11 (0.93-1.32)	0.257
Widowed	2.29 (2.01-2.62)	<0.001	1.36 (1.17-1.57)	<0.001	2.55 (2.20-2.96)	<0.001	1.45 (1.23-1.71)	<0.001
**Stage II**								
Married	Reference		Reference		Reference		Reference	
Divorced	1.19 (1.05-1.35)	0.007	1.18 (1.04-1.34)	0.013	1.19 (1.04-1.37)	0.011	1.17 (1.02-1.35)	0.025
Single	1.15 (1.02-1.29)	0.023	1.17 (1.03-1.33)	0.012	1.11 (0.97-1.26)	0.135	1.11 (0.97-1.27)	0.130
Widowed	1.53 (1.36-1.73)	<0.001	1.21 (1.06-1.39)	0.005	1.53 (1.34-1.74)	<0.001	1.24 (1.07-1.43)	0.005
**Stage III**								
Married	Reference		Reference		Reference		Reference	
Divorced	1.29 (1.16-1.44)	<0.001	1.29 (1.15-1.44)	<0.001	1.25 (1.11-1.40)	<0.001	1.24 (1.10-1.40)	<0.001
Single	1.26 (1.14-1.40)	<0.001	1.25 (1.12-1.39)	<0.001	1.26 (1.13-1.40)	<0.001	1.24 (1.11-1.39)	<0.001
Widowed	1.69 (1.49-1.91)	<0.001	1.31 (1.14-1.49)	<0.001	1.62 (1.41-1.85)	<0.001	1.27 (1.10-1.46)	0.001
**Stage IV**								
Married	Reference		Reference		Reference		Reference	
Divorced	1.16 (1.07-1.26)	<0.001	1.16 (1.07-1.27)	<0.001	1.13 (1.04-1.23)	0.005	1.13 (1.04-1.24)	0.006
Single	1.21 (1.12-1.30)	<0.001	1.23 (1.14-1.33)	<0.001	1.20 (1.11-1.30)	<0.001	1.23 (1.13-1.33)	<0.001
Widowed	1.32 (1.20-1.46)	<0.001	1.17 (1.06-1.30)	0.003	1.30 (1.18-1.44)	<0.001	1.15 (1.03-1.28)	0.011

AJCC= American Joint Committee on Cancer; OS=overall survival; CSS=cause-specific survival; TNM= tumor, node and metastasis.

### The effects of marital status on overall survival and cause-specific survival in the 5,972-matched patient cohort with esophageal cancer

Using a 1:1 propensity score matching method, we matched 2,986 unmarried patients with 2,986 married patients. As shown in Supplementary Table 3, the SD between all the baseline clinicopathological factors decreased after matching the data. Using a SD of 0.1 as a cutoff for imbalance, the distribution of sex, race, histology, tumor location, and type of therapy between the two groups reached balance (see Supplementary Table 3).

**Figure 3:**
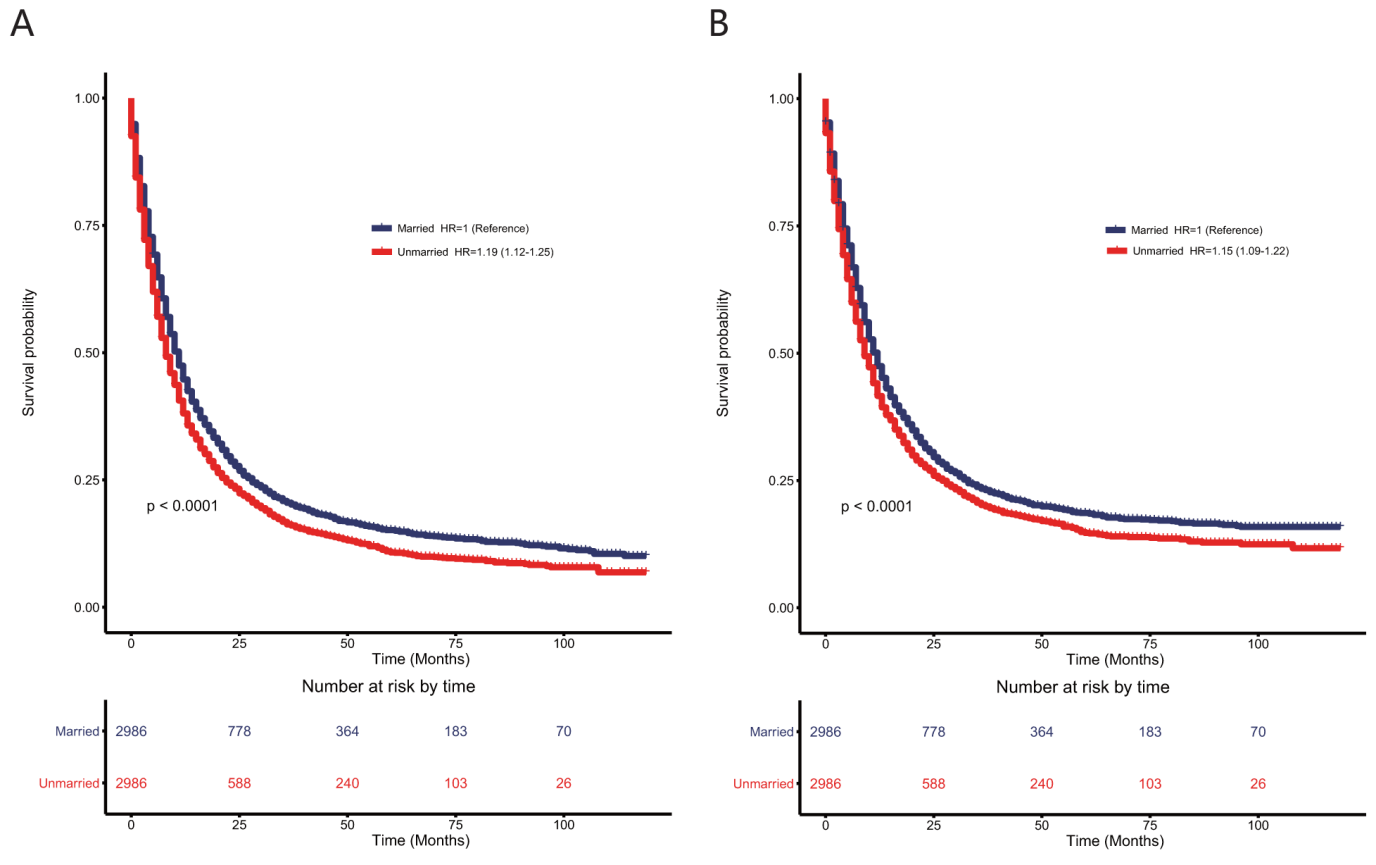
Kaplan–Meier survival plots in patients with esophageal cancers in the matched cohort with 2,986 unmarried and 2,986 married patients according to marital status **(A)** overall survival; **(B)** esophageal cancer cause-specific survival.

**Figure 4:**
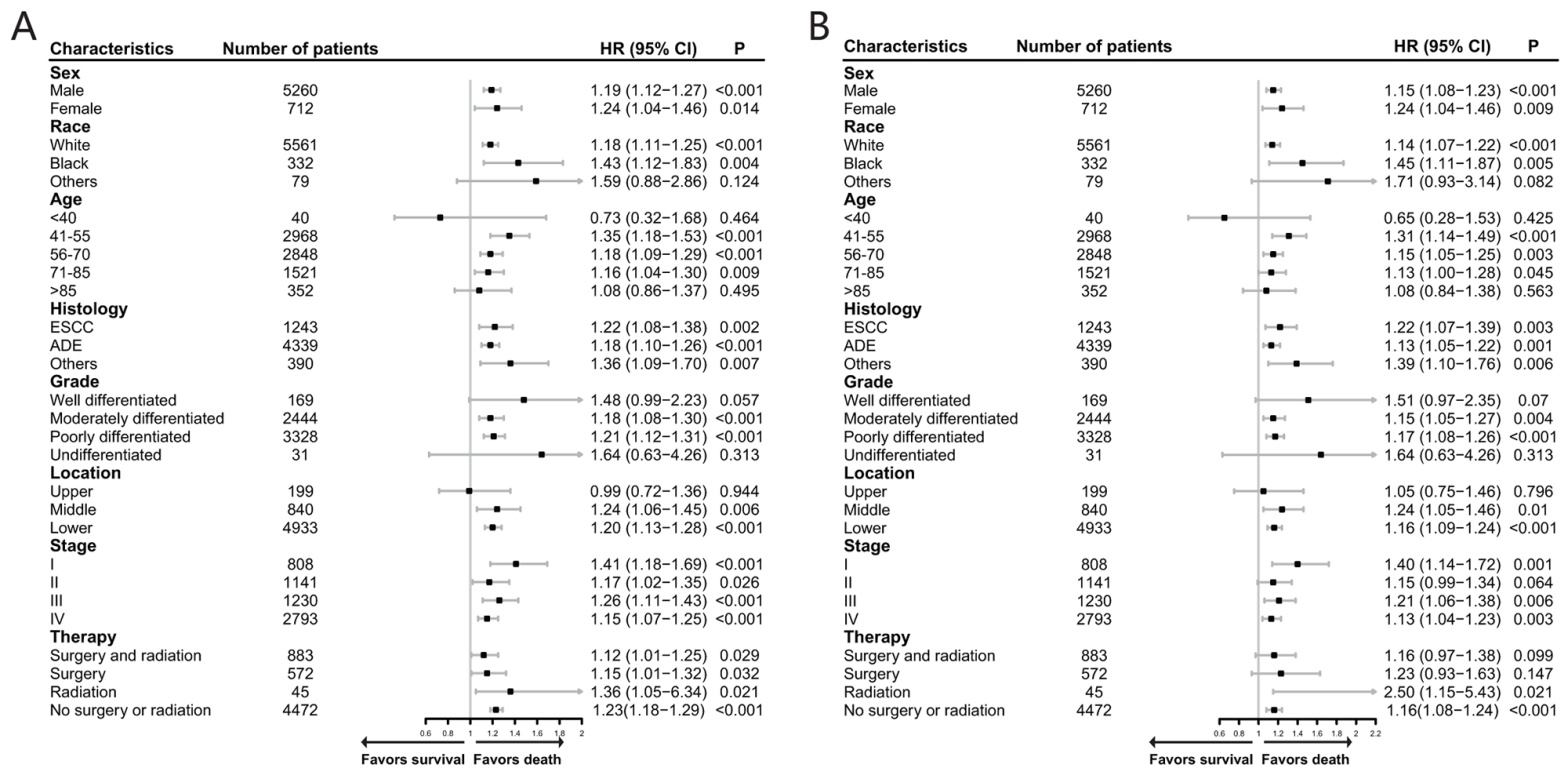
Forest plot presenting the contribution of unmarried status compared with that of married status to the survival rates of patients in the subgroups of the matched cohort according to different clinicopathological factors **(A)** overall survival; **(B)** esophageal cancer cause-specific survival. HR > 1 with P < 0.05 meant that unmarried status contributed significantly to poorer survival than married status.

As shown in the Figure 3, although the HR was not higher after matching the data than before matching the data, unmarried patients still had poorer OS (HR: 1.19; 95% CI: 1.12–1.25; P < 0.0001) and CSS (HR: 1.15; 95% CI: 1.09–1.22; P < 0.0001) than married patients in the univariate Cox analysis. Even after adjusting for these baseline characteristics using multivariate Cox regression (see Supplementary Table 4), unmarried patients still had poorer OS (HR: 1.20; 95% CI: 1.13–1.27; P < 0.0001) and CSS (HR: 1.17; 95% CI: 1.10–1.24; P < 0.0001) than married patients. To examine the credibility of our conclusions, we conducted additional sensitivity and subgroup analysis. As shown in Figure 4, most of the subgroup analyses showed that unmarried patients had poorer OS and CSS than married patients. Although no significance was reached for some subgroup analyses, such as the analyses of patients of other races for OS and patients with grade I tumors, due to the limited number of patients, there were trends indicating that unmarried status contributed to poorer OS and CSS.

## DISCUSSION

This study was the first to use a systematic PSM method to show that unmarried status was an independent risk factor contributing to poor OS and CSS in EC patients. Unmarried patients had poor OS and CSS regardless of whether the cohort was adjusted for age, sex, race, histology, tumor grade, tumor location, or type of therapy. Even in the matched cohort, unmarried status contributed to the poor survival rate of EC patients. Moreover, different types of unmarried statuses, including single, widowed, and divorced statuses, all contributed to poorer survival rates than married status. Among the unmarried patients, widowed patients were the most likely to die of EC even when adjusting for age, sex, race, tumor stage, tumor grade, tumor location, tumor histology, and type of therapy.

Multiple studies have been conducted to show that unmarried patients with gastrointestinal tumors had poorer survival rates than married patients [3, 5, 6, 14–19], but these studies included limited evidence about EC patients. Two Swedish population-based cohorts investigated the role of marital status in the survival rates of EC patients but reached contractionary conclusions. Brusselaers et al. examined 606 EC patients and divided marital status into married, remarried, never married, and previously married statuses [10]. This study found no evidence of better five-year survival rates in married patients than in unmarried patients, though this conclusion can likely be attributed to the limited number of patients included in the study. Another Swedish study involved all Swedish residents aged 30–84 years in 1990–2007 and analyzed the role of sociodemographic and geographical factors, including marital status, and produced positive results that showed that married residents had better EC survival rates than unmarried residents [9]. However, this study could not adjust for the clinicopathological factors of EC because it was based on the Causes of Death Register. Therefore, the effect of marital status on the EC survival rate should be considered cautiously. One large well-designed, comprehensive population-based study analyzed cancers associated with the ten leading causes of cancer-related death, including EC, and found that unmarried status contributed to poor survival rates in all ten cancers [3]. However, tumor grade, location of EC, tumor histology, and more detailed therapy information that may affect the survival rate of EC patients were not included for analysis in this study.

In accordance with Aizer’s study, the unmarried patients examined by the present study received less therapy than married patients. However, unmarried patients had percentages of stage I, II, III, and IV tumors that were comparable with that of married patients, with a SD of 0.049, which differed from Aizer’s study. This difference may be because metastatic tumors were considered late-stage tumors in Aizer’s study, but TNM stage was used in our study. Unlike Aizer’s study, we analyzed the role of different subdivisions of unmarried statuses in the survival rate of EC patients and found that widowed patients had the poorest survival rate of the overall EC population. In addition, we used PSM, which is an effective tool to address selection and residual biases [20, 21], to analyze relationships further to support our conclusions that being unmarried contributes to poor EC survival rates. Interestingly, widowed patients, who had the poorest survival rates, were more likely to be diagnosed at an early stage (22.89%) compared with 14.85%, 16.11%, and 13.50% in single, married, and divorced patients, respectively, which were rates similar to those found in gastric cancer patients [6]. In addition, our study explored whether the contributions of unmarried status and even the different subdivisions of unmarried statuses persisted in subgroups of EC patients with different stages of cancer. Interestingly, subgroup analysis showed that widowed patients had the poorest survival rate for stage I, II, and III EC, but single patients had the poorest survival rate for stage IV EC.

These results indicate that some latent risk factors also play an important role in the poor survival rate of unmarried patients. These risk factors include social factors, which could be explained hypothetically from psychosocial and socioeconomic perspectives. Having a support system that includes emotional support is one factor that might affect cancer survival rates from a psychosocial perspective, and financial support is one factor that might affect these rates from a socioeconomic perspective. From a socioeconomic perspective, patients with cancer usually have financial dilemmas due to the high cost of cancer treatments. Having another source of income could help lighten this financial burden [22, 23]. Without enough financial support to cover their EC treatments, some unmarried patients may refuse to receive the care they need, which was proved in our study. However, the income of a married patient’s partner could help alleviate the burden of these medical expenses.

From a psychosocial perspective, spouses could also help married patients receive the care they need and encourage married patients to be positive about the outcome of their EC, but this type of support is a privilege that unmarried patients lack. In addition, being unmarried has been associated with a higher incidence of depression among cancer patients [14, 24–26]. Studies have shown that depressive disorders could affect nearly 26% of patients, with some of these patients refusing to be treated actively, and the symptoms of this depression might even persist long after therapy or could reappear upon cancer recurrence [27, 28]. In our study, after using PSM, the differences between the tumor characteristics and the extent of treatment, such as tumor grade, therapy options, and tumor stage at diagnosis, nearly disappeared, but unmarried status still contributed to poorer prognoses. This result further supported previous results that suggested that unmarried status affects survival on a psychosocial level.

Since psychosocial support influences the prognoses of EC patients, marital status should be taken into consideration, especially widowed status, which carries the highest risk of death. Unmarried EC patients should be provided with more psychosocial care. For example, counseling could enable unmarried patients to discuss their EC diagnosis and therapy options. Meanwhile, barriers in seeking optimal care could be reduced [29]. In addition, social workers could also help unmarried patients during their initial EC diagnosis, treatment, and follow-ups with adequate companion and life assistance [30, 31].

Several limitations of our study should be discussed. First, the marital status analyzed in our study was defined at the initial diagnosis of each tumor and might have changed during follow-up periods. We could not adjust this bias because the SEER database only included information about marital status at the time of diagnosis. Second, detailed information about the patients’ therapies, such as chemotherapy and therapy quality, was not provided. Third, only legally married patients were classified as married, which meant that cohabitating patients were not recorded as married, biasing our results. According to the 2010 US Census, approximately 90 million unmarried individuals were cohabitating with other people, whereas only about 30 million (25%) lived alone. Fourth, patients with unknown clinicopathological characteristics were excluded from our analysis, causing selection bias. However, this problem was addressed to some extent by the use of PSM in our study. Fifth, our matching procedure resulted in the inclusion of only 38% (5,972/15,598) of the original cohort.

Despite the inevitable limitations of our study, our results demonstrated that unmarried status could contribute to the poor survival rates of EC patients, and these conclusions were further confirmed by PSM. Furthermore, after unmarried status was divided into three subgroups, we showed that all single, widowed, and divorced patients had poorer prognoses than married patients. Among these unmarried subgroups, widowed patients had the worst prognoses, which suggests that these patients need more counseling and comprehensive case management.

## METHODS

### Data source

Data was obtained from the SEER program, which is sponsored by the National Cancer Institute. The SEER program includes data from 18 population-based cancer registries from 1973 to 2013, which represents approximately 30% of US population. The program collects data about cancer stage, grade, therapy, incidence, and demographic information, such as age, sex, race, and marital status. The current dataset used for this analysis was based on the Incidence-SEER 18 Register Research Data + Hurricane Katrina Impacted Louisiana Cases, Nov2015 Sub (1973–2013 varying). Since the SEER database did not include personal identifying information, informed consent was not required in our study. However, permission to access SEER database was approved using the private SEER ID (13526-Nov2015). This study was approved by our institutional review board at Ren Ji Hospital.

### Inclusion and exclusion criteria

Patients with esophageal primary cancers were identified with the following inclusion criteria: 1) site recode ICD-O-3/WHO 2008 (International Classification of Diseases for Oncology, 3rd edition) was restricted to the “Esophagus”; 2) the year of diagnosis of the cancers ranged from 2004 to 2012; 3) EC was the only single cancer or was the first tumor of more than one primary tumors; 4) all relevant factors contributing to survival should be known, including age, sex, race, histology, TNM stage, tumor grade, whether surgery was performed, location of the primary cancer, and whether radiotherapy was performed; and 5) the cause of death and survival time were both known.

### Variables

The main independent variable of interest was each patient’s marital status at the initial diagnosis of the primary cancer. Marital status was redefined as married or unmarried (including single, widowed, divorced, separated, or domestic partner). For further analysis, we also divided marital status into four groups: married, divorced (including separated and divorced), single (including single, unmarried, and domestic partner) and widowed. Race was classified as White, Black, or other (including American Indian/AK Native and Asian/Pacific Islander) and excluded unknown race. Age was presented with five groups: < 40, 41–55, 56–70, 71–85, > 85. Histology was reclassified as esophageal squamous cell carcinoma (ESCC), EAC, or other since ESCC and EAC are two main types of esophageal cancers. Tumor grade was defined as well differentiated, moderately differentiated, poorly differentiated, or undifferentiated, excluding unknown grade. We divided tumor location into three groups, including the upper third of the esophagus, the middle third of the esophagus, and the lower third of the esophagus. TNM stage was defined as stage I, II, III, or IV using the AJCC Cancer Staging Manual (sixth edition). Type of therapy was defined as whether surgery or radiotherapy was performed, including no surgery or radiotherapy, only surgery, only radiotherapy, or surgery and radiotherapy, because whether surgery or radiotherapy were performed could contribute to the survival outcomes of patients.

The main outcomes were OS and CSS. CSS was defined as a net survival measure representing the survival of a specific cause of death, excluding other causes of death, according the SEER database. In this study, CSS was defined as death due to EC, with individuals who died of causes other than EC being censored.

### Statistical analysis

The clinical characteristics of the patients with EC were presented with descriptive statistics. The categorical variable was presented with the proportion of patients. The continuous variable was shown as mean ± standard deviation. A Kaplan–Meier plot was used to show OS and CSS. The comparison between patients with different marital statuses was performed using the log-rank test. In addition, HR was calculated for known prognostic factors, including age, sex, race, therapy, histology, TNM stage, tumor grade, tumor location, and marital status, using the univariate Cox proportional hazard model and the multivariate Cox proportional hazard model.

In order to reduce imbalance, we also used PSM to carry out a matched case-control analysis [20]. First, the propensity scores were calculated for marital status (married and unmarried) for each of the 15,598 patients using a nonparsimonious multivariable logistic regression model. In this model, marital status was used as the dependent variable, with all recorded variables shown in Table 1 included as covariates. Second, using an SPSS matching macro, we matched married and unmarried patients who had very similar propensity scores. Third, after matching, all baseline covariates between married and unmarried patients before and after propensity scores were matched using SD < 0.1, suggesting that these covariates between married and unmarried patients were well balanced. Fourth, we used Kaplan–Meier survival analyses to compare the OS and CSS of married and unmarried patients. In addition, the Cox univariate proportional hazards model and the Cox multivariate proportional hazards model were used to calculate the HR of marital status on OS and CSS, respectively. Fifth, sensitivity analyses and subgroup analyses were conducted using Cox multivariate proportional hazards model to determine the reliability of the association between marital status and OS or CSS [32, 33].

All statistical tests were evaluated using a two-tailed 95% CI, and two-side P < 0.05 was set for statistical significance. All data analyses were performed using the statistical software package SPSS for Windows, version 17 (SPSS Inc, Chicago, IL, USA).

## SUPPLEMENTARY MATERIALS TABLES











## Abbreviations

EC: esophageal cancer
SEER: the surveillance, epidemiology and end results
OS: overall survival
CSS: cancer specific survival
HR: hazard ratio
PSM: propensity score matching
TNM: tumor, node and metastasis
SD: standard difference
CI: confidence level
ESCC: esophageal squamous cell carcinoma
EAC: esophageal adenocarcinoma
