# Supplementary material for: The influence of marital status on the survival of patients with esophageal cancer: a population-based, propensity-matched study
Source: Oncotarget. 2017 Jul 22;8(37):62261–73. doi: 10.18632/oncotarget.19446 (PMC5617503; doi:10.18632/oncotarget.19446)
Supplement: Supplementary file 1 [file oncotarget-08-62261-s001.pdf]

The influence of marital status on the survival of patients with esophageal cancer: a population-based, propensity-matched study

SUPPLEMENTARY MATERIALS

Supplementary Table 1: Univariate and multivariate cox regression analysis of unmarried status compared with married status on OS and esophageal cancer CSS based on different subgroups of baseline characteristics in 15598 unmatched cohort with esophageal cancer.

See Supplementary File 1

Supplementary Table 2: Univariate and multivariate cox regression analysis of divorced, single and widowed status compared with married status on OS and esophageal cancer CSS based on different subgroups of baseline characteristics in 15598 unmatched cohort with esophageal cancer.

See Supplementary File 2

Supplementary Table 3: Standard difference of characteristics before matching and after matching respectively.

See Supplementary File 3

Supplementary Table 4: Univariate and multivariate cox regression analysis of unmarried status compared with married status on OS and esophageal cancer CSS based on different subgroups of baseline characteristics in the 5972 matched cohort with esophageal cancer.

See Supplementary File 4
