# Supplementary material for: The influence of marital status on the survival of patients with esophageal cancer: a population-based, propensity-matched study
Source: Oncotarget. 2017 Jul 22;8(37):62261–73. doi: 10.18632/oncotarget.19446 (PMC5617503; doi:10.18632/oncotarget.19446)
Supplement: Supplementary file 3 [file oncotarget-08-62261-s003.docx]

**Supplementary Table 2: Univariate and multivariate cox regression analysis of divorced, single and widowed status compared with married status on OS and esophageal cancer CSS based on different subgroups of baseline characteristics in 15598 unmatched cohort with esophageal cancer**

| Variable | OS | | | | CSS | | | |
| --- | --- | --- | --- | --- | --- | --- | --- | --- |
|  | Univariate analysis | | Multivariate analysis | | Univariate analysis | | Multivariate analysis | |
|  | HR (95% CI) | P | HR (95% CI) | P | HR (95% CI) | P | HR (95% CI) | P |
| **Sex** |  |  |  |  |  |  |  |  |
| **Male** |  |  |  |  |  |  |  |  |
| Married | Reference |  | Reference |  | Reference |  | Reference |  |
| Divorced | 1.27 (1.20-1.35) | <0.001 | 1.22 (1.15-1.30) | <0.001 | 1.24 (1.16-1.33) | <0.001 | 1.19 (1.11-1.27) | <0.001 |
| Single | 1.22 (1.15-1.29) | <0.001 | 1.21 (1.14-1.28) | <0.001 | 1.20 (1.13-1.28) | <0.001 | 1.18 (1.11-1.26) | <0.001 |
| Widowed | 1.53 (1.42-1.66) | <0.001 | 1.25 (1.15-1.35) | <0.001 | 1.48 (1.36-1.61) | <0.001 | 1.24 (1.14-1.36) | <0.001 |
| **Female** |  |  |  |  |  |  |  |  |
| Married | Reference |  | Reference |  | Reference |  | Reference |  |
| Divorced | 1.14 (1.00-1.30) | 0.048 | 1.13 (0.99-1.29) | 0.080 | 1.15 (1.00-1.32) | 0.047 | 1.14 (0.99-1.31) | 0.075 |
| Single | 1.24 (1.10-1.40) | <0.001 | 1.23 (1.09-1.40) | 0.001 | 1.26 (1.10-1.43) | 0.001 | 1.25 (1.09-1.43) | 0.001 |
| Widowed | 1.50 (1.35-1.66) | <0.001 | 1.23 (1.11-1.37) | <0.001 | 1.49 (1.34-1.66) | <0.001 | 1.24 (1.10-1.39) | <0.001 |
| **Race** |  |  |  |  |  |  |  |  |
| **White** |  |  |  |  |  |  |  |  |
| Married | Reference |  | Reference |  | Reference |  | Reference |  |
| Divorced | 1.23 (1.15-1.30) | <0.001 | 1.22 (1.14-1.29) | <0.001 | 1.20 (1.13-1.28) | <0.001 | 1.19 (1.11-1.26) | <0.001 |
| Single | 1.12 (1.05-1.18) | <0.001 | 1.17 (1.10-1.24) | <0.001 | 1.11 (1.04-1.18) | 0.001 | 1.15 (1.08-1.23) | <0.001 |
| Widowed | 1.46 (1.37-1.56) | <0.001 | 1.22 (1.14-1.31) | <0.001 | 1.43 (1.34-1.53) | <0.001 | 1.23 (1.14-1.32) | <0.001 |
| **Black** |  |  |  |  |  |  |  |  |
| Married | Reference |  | Reference |  | Reference |  | Reference |  |
| Divorced | 1.19 (1.00-1.40) | 0.044 | 1.13 (0.96-1.34) | 0.146 | 1.17 (0.98-1.40) | 0.085 | 1.10 (0.92-1.31) | 0.310 |
| Single | 1.37 (1.19-1.56) | <0.001 | 1.33 (1.16-1.53) | <0.001 | 1.34 (1.19-1.56) | <0.001 | 1.27 (1.10-1.48) | <0.001 |
| Widowed | 1.42 (1.19-1.69) | <0.001 | 1.41 (1.17-1.71) | <0.001 | 1.32 (1.09-1.61) | 0.004 | 1.32 (1.08-1.62) | 0.007 |
| **Other race** |  |  |  |  |  |  |  |  |
| Married | Reference |  | Reference |  | Reference |  | Reference |  |
| Divorced | 1.08 (0.80-1.46) | 0.606 | 1.57 (1.16-2.13) | 0.004 | 1.10 (0.80-1.52) | 0.549 | 1.61 (1.16-2.24) | 0.004 |
| Single | 1.39 (1.10-1.77) | 0.007 | 1.44 (1.13-1.83) | 0.004 | 1.49 (1.16-1.91) | 0.002 | 1.52 (1.18-1.96) | 0.001 |
| Widowed | 1.32 (1.02-1.70) | 0.032 | 1.42 (1.07-1.89) | 0.017 | 1.37 (1.05-1.78) | 0.022 | 1.48 (1.09-2.00) | 0.012 |
| **Age** |  |  |  |  |  |  |  |  |
| **<40** |  |  |  |  |  |  |  |  |
| Married | Reference |  | Reference |  | Reference |  | Reference |  |
| Divorced | 1.08 (0.59-2.00) | 0.606 | 0.84 (0.44-1.59) | 0.590 | 1.16 (0.63-2.14) | 0.632 | 0.92 (0.48-1.73) | 0.788 |
| Single | 1.21 (0.88-1.67) | 0.244 | 0.99 (0.69-1.42) | 0.948 | 1.18 (0.84-1.64) | 0.244 | 0.98 (0.67-1.42) | 0.900 |
| Widowed | 2.81 (0.68-11.51) | 0.152 | 1.18 (0.25-5.49) | 0.836 | 2.91 (0.71-11.96) | 0.138 | 1.00 (0.21-4.84) | 0.998 |
| **41-55** |  |  |  |  |  |  |  |  |
| Married | Reference |  | Reference |  | Reference |  | Reference |  |
| Divorced | 1.44 (1.28-1.63) | <0.001 | 1.32 (1.17-1.49) | <0.001 | 1.40 (1.24-1.59) | <0.001 | 1.29 (1.14-1.46) | <0.001 |
| Single | 1.43 (1.29-1.57) | <0.001 | 1.35 (1.22-1.49) | <0.001 | 1.38 (1.25-1.53) | <0.001 | 1.32 (1.19-1.47) | <0.001 |
| Widowed | 1.54 (1.13-2.11) | 0.006 | 1.27 (0.74-0.95) | 0.134 | 1.45 (1.04-2.03) | 0.027 | 1.22 (0.87-1.71) | 0.247 |
| **56-70** |  |  |  |  |  |  |  |  |
| Married | Reference |  | Reference |  | Reference |  | Reference |  |
| Divorced | 1.23 (1.14-1.33) | <0.001 | 1.17 (1.08-1.26) | <0.001 | 1.20 (1.11-1.30) | <0.001 | 1.13 (1.04-1.23) | <0.001 |
| Single | 1.25 (1.16-1.34) | <0.001 | 1.15 (1.07-1.25) | <0.001 | 1.24 (1.14-1.34) | <0.001 | 1.15 (1.05-1.24) | <0.001 |
| Widowed | 1.28 (1.14-1.43) | <0.001 | 1.26 (1.12-1.42) | <0.001 | 1.25 (1.10-1.41) | <0.001 | 1.23 (1.09-1.40) | <0.001 |
| **71-85** |  |  |  |  |  |  |  |  |
| Married | Reference |  | Reference |  | Reference |  | Reference |  |
| Divorced | 1.29 (1.15-1.45) | <0.001 | 1.27 (1.13-1.43) | <0.001 | 1.26 (1.11-1.43) | <0.001 | 1.24 (1.09-1.41) | <0.001 |
| Single | 1.17 (1.03-1.31) | 0.012 | 1.13 (1.00-1.28) | 0.045 | 1.14 (1.00-1.30) | 0.047 | 1.11 (0.97-1.27) | 0.045 |
| Widowed | 1.18 (1.08-1.28) | <0.001 | 1.15 (1.05-1.26) | 0.002 | 1.19 (1.08-1.30) | <0.001 | 1.14 (1.04-1.26) | 0.002 |
| **>85** |  |  |  |  |  |  |  |  |
| Married | Reference |  | Reference |  | Reference |  | Reference |  |
| Divorced | 0.96 (0.62-1.48) | 0.846 | 0.95 (0.61-1.49) | 0.825 | 0.95 (0.59-1.54) | 0.831 | 0.96 (0.58-1.56) | 0.855 |
| Single | 1.10 (0.81-1.50) | 0.527 | 1.25 (0.90-1.73) | 0.182 | 1.00 (0.70-1.42) | 0.994 | 1.13 (0.78-1.63) | 0.514 |
| Widowed | 1.17 (0.99-1.39) | 0.066 | 1.23 (1.02-1.48) | 0.032 | 1.21 (1.01-1.45) | 0.038 | 1.25 (1.03-1.53) | 0.027 |
| **Histology** |  |  |  |  |  |  |  |  |
| **ESCC** |  |  |  |  |  |  |  |  |
| Married | Reference |  | Reference |  | Reference |  | Reference |  |
| Divorced | 1.29 (1.18-1.42) | <0.001 | 1.31 (1.18-1.44) | <0.001 | 1.28 (1.15-1.42) | <0.001 | 1.29 (1.16-1.43) | <0.001 |
| Single | 1.29 (1.18-1.40) | <0.001 | 1.27 (1.16-1.39) | <0.001 | 1.29 (1.18-1.41) | <0.001 | 1.27 (1.16-1.39) | <0.001 |
| Widowed | 1.36 (1.24-1.50) | <0.001 | 1.26 (1.14-1.40) | <0.001 | 1.35 (1.22-1.49) | <0.001 | 1.27 (1.14-1.42) | <0.001 |
| **EAC** |  |  |  |  |  |  |  |  |
| Married | Reference |  | Reference |  | Reference |  | Reference |  |
| Divorced | 1.18 (1.10-1.27) | <0.001 | 1.15 (1.07-1.24) | <0.001 | 1.16 (1.08-1.26) | <0.001 | 1.13 (1.04-1.22) | 0.003 |
| Single | 1.14 (1.06-1.22) | <0.001 | 1.17 (1.09-1.26) | <0.001 | 1.12 (1.041.21) | 0.003 | 1.14 (1.06-1.23) | 0.001 |
| Widowed | 1.52 (1.41-1.65) | <0.001 | 1.28 (1.17-1.39) | <0.001 | 1.48 (1.36-1.61) | <0.001 | 1.26 (1.15-1.38) | <0.001 |
| **Others** |  |  |  |  |  |  |  |  |
| Married | Reference |  | Reference |  | Reference |  | Reference |  |
| Divorced | 1.20 (0.99-1.45) | 0.060 | 1.18 (0.97-1.43) | 0.094 | 1.18 (0.97-1.44) | 0.103 | 1.13 (0.92-1.39) | 0.228 |
| Single | 1.20 (1.00-1.44) | 0.053 | 1.25 (1.03-1.52) | 0.021 | 1.23 (1.02-1.50) | 0.029 | 1.27 (1.04-1.55) | 0.018 |
| Widowed | 1.35 (1.11-1.63) | 0.002 | 1.20 (0.97-1.49) | 0.087 | 1.38 (1.13-1.68) | 0.002 | 1.25 (1.00-1.56) | 0.049 |
| **Grade** |  |  |  |  |  |  |  |  |
| **Well differentiated** |  |  |  |  |  |  |  |  |
| Married | Reference |  | Reference |  | Reference |  | Reference |  |
| Divorced | 1.45 (1.12-1.90) | 0.006 | 1.34 (1.02-1.76) | 0.034 | 1.39 (1.03-1.89) | 0.032 | 1.26 (0.93-1.72) | 0.139 |
| Single | 1.18 (0.92-1.2) | 0.197 | 1.02 (0.78-1.34) | 0.862 | 1.24 (0.94-1.64) | 0.126 | 1.07 (0.79-1.44) | 0.665 |
| Widowed | 2.37 (1.84-3.06) | <0.001 | 1.69 (1.26-2.26) | <0.001 | 2.53 (1.92-3.33) | <0.001 | 1.80 (1.31-2.48) | <0.001 |
| **Moderately differentiated** | |  |  |  |  |  |  |  |
| Married | Reference |  | Reference |  | Reference |  | Reference |  |
| Divorced | 1.25 (1.14-1.36) | <0.001 | 1.17 (1.07-1.28) | 0.001 | 1.26 (1.14-1.38) | <0.001 | 1.17 (1.06-1.28) | 0.002 |
| Single | 1.30 (1.20-1.41) | <0.001 | 1.22 (1.12-1.32) | <0.001 | 1.30 (1.20-1.41) | <0.001 | 1.20 (1.10-1.31) | <0.001 |
| Widowed | 1.53 (1.40-1.68) | <0.001 | 1.26 (1.14-1.39) | <0.001 | 1.52 (1.38-1.68) | <0.001 | 1.28 (1.15-1.43) | <0.001 |
| **Poorly differentiated** | |  |  |  |  |  |  |  |
| Married | Reference |  | Reference |  | Reference |  | Reference |  |
| Divorced | 1.22 (1.13-1.32) | <0.001 | 1.21 (1.12-1.30) | <0.001 | 1.19 (1.10-1.29) | <0.001 | 1.17 (1.07-1.26) | <0.001 |
| Single | 1.18 (1.10-1.27) | <0.001 | 1.22 (1.13-1.31) | <0.001 | 1.17 (1.09-1.26) | <0.001 | 1.20 (1.11-1.29) | <0.001 |
| Widowed | 1.40 (1.29-1.52) | <0.001 | 1.22 (1.12-1.34) | <0.001 | 1.36 (1.24-1.48) | <0.001 | 1.21 (1.10-1.32) | <0.001 |
| **Undifferentiated** |  |  |  |  |  |  |  |  |
| Married | Reference |  | Reference |  | Reference |  | Reference |  |
| Divorced | 1.69 (1.01-2.50) | 0.044 | 1.60 (1.00-2.57) | 0.051 | 1.56 (0.97-2.51) | 0.066 | 1.58 (0.96-2.59) | 0.070 |
| Single | 1.33 (0.93-1.92) | 0.123 | 1.44 (0.98-2.10) | 0.060 | 1.35 (0.93-1.96) | 0.120 | 1.47 (1.00-2.17) | 0.051 |
| Widowed | 1.12 (0.78-1.60) | 0.542 | 1.30 (0.85-1.97) | 0.224 | 1.04 (0.71-1.53) | 0.850 | 1.31 (0.84-2.06) | 0.236 |
| **Location** |  |  |  |  |  |  |  |  |
| **Upper third of esophagus** | |  |  |  |  |  |  |  |
| Married | Reference |  | Reference |  | Reference |  | Reference |  |
| Divorced | 1.24 (1.02-1.51) | 0.031 | 1.20 (0.98-1.47) | 0.077 | 1.32 (1.07-1.62) | 0.009 | 1.26 (1.02-1.57) | 0.033 |
| Single | 1.15 (0.96-1.38) | 0.119 | 1.08 (0.90-1.31) | 0.409 | 1.15 (0.94-1.39) | 0.169 | 1.06 (0.86-1.30) | 0.591 |
| Widowed | 1.13 (0.92-1.39) | 0.245 | 1.07 (0.86-1.34) | 0.552 | 1.17 (0.94-1.46) | 0.158 | 1.15 (0.90-1.46) | 0.256 |
| **Middle third of esophagus** | |  |  |  |  |  |  |  |
| Married | Reference |  | Reference |  | Reference |  | Reference |  |
| Divorced | 1.24 (1.10-1.39) | <0.001 | 1.32 (1.17-1.48) | <0.001 | 1.21 (1.07-1.38) | 0.003 | 1.29 (1.14-1.47) | <0.001 |
| Single | 1.27 (1.14-1.41) | <0.001 | 1.31 (1.17-1.46) | <0.001 | 1.27 (1.14-1.42) | <0.001 | 1.31 (1.16-1.47) | <0.001 |
| Widowed | 1.39 (1.24-1.56) | <0.001 | 1.30 (1.14-1.48) | <0.001 | 1.38 (1.22-1.56) | <0.001 | 1.31 (1.14-1.51) | <0.001 |
| **Lower third of esophagus** | |  |  |  |  |  |  |  |
| Married | Reference |  | Reference |  | Reference |  | Reference |  |
| Divorced | 1.22 (1.14-1.30) | <0.001 | 1.17 (1.09-1.25) | <0.001 | 1.19 (1.10-1.27) | <0.001 | 1.13 (1.05-1.21) | 0.001 |
| Single | 1.18 (1.11-1.26) | <0.001 | 1.21 (1.13-1.28) | <0.001 | 1.18 (1.10-1.25) | <0.001 | 1.19 (1.11-1.27) | <0.001 |
| Widowed | 1.53 (1.42-1.64) | <0.001 | 1.27 (1.17-1.37) | <0.001 | 1.48 (1.37-1.60) | <0.001 | 1.25 (1.15-1.36) | <0.001 |
| **TNM Stage** |  |  |  |  |  |  |  |  |
| **Stage I** |  |  |  |  |  |  |  |  |
| Married | Reference |  | Reference |  | Reference |  | Reference |  |
| Divorced | 1.41 (1.19-1.66) | <0.001 | 1.37 (1.16-1.63) | <0.001 | 1.47 (1.22-1.78) | <0.001 | 1.37 (1.13-1.67) | <0.001 |
| Single | 1.21 (1.05-1.41) | 0.011 | 1.14 (0.98-1.32) | 0.100 | 1.24 (1.05-1.48) | 0.013 | 1.11 (0.93-1.32) | 0.257 |
| Widowed | 2.29 (2.01-2.62) | <0.001 | 1.36 (1.17-1.57) | <0.001 | 2.55 (2.20-2.96) | <0.001 | 1.45 (1.23-1.71) | <0.001 |
| **Stage II** |  |  |  |  |  |  |  |  |
| Married | Reference |  | Reference |  | Reference |  | Reference |  |
| Divorced | 1.19 (1.05-1.35) | 0.007 | 1.18 (1.04-1.34) | 0.013 | 1.19 (1.04-1.37) | 0.011 | 1.17 (1.02-1.35) | 0.025 |
| Single | 1.15 (1.02-1.29) | 0.023 | 1.17 (1.03-1.33) | 0.012 | 1.11 (0.97-1.26) | 0.135 | 1.11 (0.97-1.27) | 0.130 |
| Widowed | 1.53 (1.36-1.73) | <0.001 | 1.21 (1.06-1.39) | 0.005 | 1.53 (1.34-1.74) | <0.001 | 1.24 (1.07-1.43) | 0.005 |
| **Stage III** |  |  |  |  |  |  |  |  |
| Married | Reference |  | Reference |  | Reference |  | Reference |  |
| Divorced | 1.29 (1.16-1.44) | <0.001 | 1.29 (1.15-1.44) | <0.001 | 1.25 (1.11-1.40) | <0.001 | 1.24 (1.10-1.40) | <0.001 |
| Single | 1.26 (1.14-1.40) | <0.001 | 1.25 (1.12-1.39) | <0.001 | 1.26 (1.13-1.40) | <0.001 | 1.24 (1.11-1.39) | <0.001 |
| Widowed | 1.69 (1.49-1.91) | <0.001 | 1.31 (1.14-1.49) | <0.001 | 1.62 (1.41-1.85) | <0.001 | 1.27 (1.10-1.46) | 0.001 |
| **Stage IV** |  |  |  |  |  |  |  |  |
| Married | Reference |  | Reference |  | Reference |  | Reference |  |
| Divorced | 1.16 (1.07-1.26) | <0.001 | 1.16 (1.07-1.27) | <0.001 | 1.13 (1.04-1.23) | 0.005 | 1.13 (1.04-1.24) | 0.006 |
| Single | 1.21 (1.12-1.30) | <0.001 | 1.23 (1.14-1.33) | <0.001 | 1.20 (1.11-1.30) | <0.001 | 1.23 (1.13-1.33) | <0.001 |
| Widowed | 1.32 (1.20-1.46) | <0.001 | 1.17 (1.06-1.30) | 0.003 | 1.30 (1.18-1.44) | <0.001 | 1.15 (1.03-1.28) | 0.011 |
| **Therapy** |  |  |  |  |  |  |  |  |
| **Surgery and radiotherapy** | |  |  |  |  |  |  |  |
| Married | Reference |  | Reference |  | Reference |  | Reference |  |
| Divorced | 1.11 (0.96-1.29) | 0.149 | 1.16 (1.00-1.34) | 0.053 | 1.06 (0.90-1.24) | 0.465 | 1.16 (1.00-1.34) | 0.053 |
| Single | 1.01 (0.88-1.17) | 0.859 | 1.09 (0.94-1.27) | 0.234 | 0.98 (0.84-1.15) | 0.804 | 1.09 (0.94-1.27) | 0.234 |
| Widowed | 1.12 (0.90-1.38) | 0.306 | 1.12 (0.90-1.40) | 0.307 | 1.12 (0.90-1.38) | 0.306 | 1.12 (0.90-1.40) | 0.307 |
| **Only surgery** | |  |  |  |  |  |  |  |
| Married | Reference |  | Reference |  | Reference |  | Reference |  |
| Divorced | 1.24 (1.03-1.50) | 0.026 | 1.31 (1.08-1.59) | 0.006 | 1.21 (0.97-1.50) | 0.089 | 1.09 (1.93-1.28) | 0.289 |
| Single | 1.03 (0.87-1.23) | 0.742 | 1.04 (0.87-1.25) | 0.677 | 1.02 (0.84-1.25) | 0.811 | 1.04 (0.88-1.22) | 0.663 |
| Widowed | 1.49 (1.22-1.82) | <0.001 | 1.16 (0.94-1.43) | 0.179 | 1.42 (1.22-1.82) | 0.003 | 1.12 (0.88-1.44) | 0.346 |
| **Only radiotherapy** |  |  |  |  |  |  |  |  |
| Married | Reference |  | Reference |  | Reference |  | Reference |  |
| Divorced | 1.03 (0.71-1.49) | 0.872 | 1.32 (0.90-1.93) | 0.158 | 1.06 (0.72-1.54) | 0.770 | 1.37 (0.92-2.04) | 0.116 |
| Single | 1.08 (0.79-1.47) | 0.640 | 1.31 (0.93-1.83) | 0.119 | 1.20 (0.87-1.64) | 0.265 | 1.47 (1.05-2.07) | 0.027 |
| Widowed | 1.35 (0.88-2.07) | 0.170 | 1.54 (0.97-2.44) | 0.070 | 1.41 (0.91-2.18) | 0.126 | 1.60 (1.00-2.58) | 0.052 |
| **No surgery or radiotherapy** | |  |  |  |  |  |  |  |
| Married | Reference |  | Reference |  | Reference |  | Reference |  |
| Divorced | 1.13 (1.06-1.20) | <0.001 | 1.20 (1.12-1.28) | <0.001 | 1.11 (1.03-1.18) | <0.001 | 1.18 (1.10-1.26) | <0.001 |
| Single | 1.13 (1.07-1.20) | <0.001 | 1.24 (1.17-1.32) | <0.001 | 1.12 (1.05-1.19) | <0.001 | 1.23 (1.15-1.31) | <0.001 |
| Widowed | 1.18 (1.10-1.25) | <0.001 | 1.26 (1.18-1.36) | <0.001 | 1.15 (1.07-1.23) | <0.001 | 1.26 (1.17-1.36) | <0.001 |

OS=overall survival; CSS=cause-specific survival; ESCC=esophageal squamous cell carcinoma; EAC=esophageal adenocarcinoma; TNM= tumor, node and metastasis.
