# Supplementary material for: The influence of marital status on the survival of patients with esophageal cancer: a population-based, propensity-matched study
Source: Oncotarget. 2017 Jul 22;8(37):62261–73. doi: 10.18632/oncotarget.19446 (PMC5617503; doi:10.18632/oncotarget.19446)
Supplement: Supplementary file 4 [file oncotarget-08-62261-s004.docx]

**Supplementary Table 3: Standard difference of characteristics before matching and after matching respectively**

| Variable | Before matching | | | After matching | | |
| --- | --- | --- | --- | --- | --- | --- |
|  | Married | Unmarried | Standard difference | Married | Unmarried | Standard difference |
| **Age (Mean±SD)** | 65.23±10.71 | 64.96±12.11 | 0.023 | 65.10±11.05 | 65.03±11.05 | 0.007 |
| **Sex** |  |  | 0.407 |  |  | 0.002 |
| Male | 8093 (87.22%) | 4488 (71.02%) |  | 2629 (88.04%) | 2631 (88.11%) |  |
| Female | 1186 (12.78%) | 1831 (28.98%) |  | 357 (11.96%) | 355 (11.89%) |  |
| **Race** |  |  | 0.396 |  |  | 0.074 |
| White | 8297 (89.42%) | 4944 (78.24%) |  | 2791 (93.47%) | 2770 (92.77%) |  |
| Black | 480 (5.17%) | 1103 (17.46%) |  | 147 (4.92%) | 185 (6.20%) |  |
| Other | 502 (5.41%) | 272 (4.30%) |  | 48 (1.61%) | 31 (1.04%) |  |
| **Grade** |  |  | 0.079 |  |  | 0.015 |
| Well differentiated | 507 (5.46%) | 337 (5.33%) |  | 88 (2.95%) | 81 (2.71%) |  |
| Moderately differentiated | 3592 (38.71%) | 2684 (42.48%) |  | 1221 (40.89%) | 1223 (40.96%) |  |
| Poorly differentiated | 5002 (53.91%) | 3170 (50.17%) |  | 1661 (55.63%) | 1667 (55.83%) |  |
| Undifferentiated | 178 (1.92%) | 128 (2.03%) |  | 16 (0.54%) | 15 (0.50%) |  |
| **TNM Stage** |  |  | 0.049 |  |  | 0.018 |
| Stage I | 1495 (16.11%) | 1053 (16.67%) |  | 407 (13.63%) | 401 (13.43%) |  |
| Stage II | 1949 (21.00%) | 1368 (21.65%) |  | 575 (19.26%) | 566 (18.96%) |  |
| Stage III | 2299 (24.78%) | 1434 (22.69%) |  | 621 (20.80%) | 609 (20.40%) |  |
| Stage IV | 3536 (38.11%) | 2464 (38.99%) |  | 1383 (46.32%) | 1410 (47.22%) |  |
| **Location** |  |  | 0.286 |  |  | 0.011 |
| Upper third of esophagus | 481 (5.18%) | 572 (9.05%) |  | 102 (3.42%) | 97 (3.25%) |  |
| Middle third of esophagus | 1595 (17.19%) | 1591 (25.18%) |  | 417 (13.97%) | 423 (14.17%) |  |
| Lower third of esophagus | 7203 (77.63%) | 4156 (65.77%) |  | 2467 (82.62%) | 2466 (82.59%) |  |
| **Therapy** |  |  | 0.289 |  |  | 0.004 |
| No therapy | 5608 (60.44%) | 4639 (73.41%) |  | 2236 (74.88%) | 2236 (74.88%) |  |
| Only surgery | 1393 (15.01%) | 714 (11.30%) |  | 286 (9.58%) | 286 (9.58%) |  |
| Only radiotherapy | 224 (2.41%) | 135 (2.14%) |  | 23 (0.77%) | 22 (0.74%) |  |
| Surgery + radiotherapy | 2054 (22.14%) | 831 (13.15%) |  | 441 (14.77%) | 442 (14.80%) |  |
| **Histology** |  |  | 0.336 |  |  | 0.011 |
| ESCC | 2231 (24.04%) | 2494 (39.47%) |  | 615 (20.60%) | 628 (21.03%) |  |
| EAC | 6193 (66.74%) | 3337 (52.81%) |  | 2176 (72.87%) | 2163 (72.44%) |  |
| Others | 855 (9.21%) | 488 (7.72%) |  | 195 (6.53%) | 195 (6.53%) |  |

ESCC=esophageal squamous cell carcinoma; EAC=esophageal adenocarcinoma; TNM= tumor, node and metastasis.
