# Supplementary material for: The influence of marital status on the survival of patients with esophageal cancer: a population-based, propensity-matched study
Source: Oncotarget. 2017 Jul 22;8(37):62261–73. doi: 10.18632/oncotarget.19446 (PMC5617503; doi:10.18632/oncotarget.19446)
Supplement: Supplementary file 5 [file oncotarget-08-62261-s005.docx]

**Supplementary Table 4: Univariate and multivariate cox regression analysis of unmarried status compared with married status on OS and esophageal cancer CSS based on different subgroups of baseline characteristics in the 5972 matched cohort with esophageal cancer**

| Variable | OS | | | | CSS | | | |
| --- | --- | --- | --- | --- | --- | --- | --- | --- |
|  | Univariate analysis | | Multivariate analysis | | Univariate analysis | | Multivariate analysis | |
|  | HR(95% CI) | P | HR(95% CI) | P | HR(95% CI) | P | HR(95% CI) | P |
| **Sex** |  |  |  |  |  |  |  |  |
| **Male** |  |  |  |  |  |  |  |  |
| Married | Reference |  | Reference |  | Reference |  | Reference |  |
| Unmarried | 1.16 (1.09-1.23) | <0.001 | 1.19 (1.12-1.27) | <0.001 | 1.11 (1.04-1.19) | 0.001 | 1.15 (1.08-1.23) | <0.001 |
| **Female** |  |  |  |  |  |  |  |  |
| Married | Reference |  | Reference |  | Reference |  | Reference |  |
| Unmarried | 1.19 (1.00-1.41) | 0.042 | 1.24 (1.04-1.46) | 0.014 | 1.22 (1.03-1.45) | 0.025 | 1.26 (1.05-1.50) | 0.009 |
| **Race** |  |  |  |  |  |  |  |  |
| **White** |  |  |  |  |  |  |  |  |
| Married | Reference |  | Reference |  | Reference |  | Reference |  |
| Unmarried | 1.14 (1.08-1.21) | <0.001 | 1.18 (1.11-1.25) | <0.001 | 1.11 (1.04-1.18) | <0.001 | 1.14 (1.07-1.22) | <0.001 |
| **Black** |  |  |  |  |  |  |  |  |
| Married | Reference |  | Reference |  | Reference |  | Reference |  |
| Unmarried | 1.31 (1.03-1.67) | 0.026 | 1.43 (1.12-1.83) | 0.004 | 1.35 (1.05-1.74) | 0.020 | 1.45 (1.11-1.87) | 0.005 |
| **Other race** |  |  |  |  |  |  |  |  |
| Married | Reference |  | Reference |  | Reference |  | Reference |  |
| Unmarried | 1.53 (0.90-2.61) | 0.119 | 1.59 (0.88-2.86) | 0.124 | 1.83 (1.05-3.21) | 0.033 | 1.71 (0.93-3.14) | 0.082 |
| **Age** |  |  |  |  |  |  |  |  |
| **<40** |  |  |  |  |  |  |  |  |
| Married | Reference |  | Reference |  | Reference |  | Reference |  |
| Unmarried | 0.81 (0.39-1.67) | 0.562 | 0.73 (0.32-1.68) | 0.464 | 0.74 (0.35-1.55) | 0.425 | 0.65 (0.28-1.53) | 0.325 |
| **41-55** |  |  |  |  |  |  |  |  |
| Married | Reference |  | Reference |  | Reference |  | Reference |  |
| Unmarried | 1.26 (1.11-1.44) | <0.001 | 1.35 (1.18-1.53) | <0.001 | 1.22 (1.07-1.40) | 0.003 | 1.31 (1.14-1.49) | <0.001 |
| **56-70** |  |  |  |  |  |  |  |  |
| Married | Reference |  | Reference |  | Reference |  | Reference |  |
| Unmarried | 1.13 (1.04-1.23) | 0.004 | 1.18 (1.09-1.29) | <0.001 | 1.10 (1.00-1.20) | 0.043 | 1.15 (1.05-1.25) | 0.003 |
| **71-85** |  |  |  |  |  |  |  |  |
| Married | Reference |  | Reference |  | Reference |  | Reference |  |
| Unmarried | 1.16 (1.04-1.29) | 0.010 | 1.16 (1.04-1.30) | 0.009 | 1.13 (1.00-1.27) | 0.045 | 1.13 (1.00-1.28) | 0.045 |
| **>85** |  |  |  |  |  |  |  |  |
| Married | Reference |  | Reference |  | Reference |  | Reference |  |
| Unmarried | 1.09 (0.87-1.38) | 0.457 | 1.08 (0.86-1.37) | 0.495 | 1.09 (0.85-1.40) | 0.457 | 1.08 (0.84-1.38) | 0.563 |
| **Histology** |  |  |  |  |  |  |  |  |
| **ESCC** |  |  |  |  |  |  |  |  |
| Married | Reference |  | Reference |  | Reference |  | Reference |  |
| Unmarried | 1.19 (1.05-1.35) | 0.005 | 1.22 (1.08-1.38) | 0.002 | 1.19 (1.04-1.35) | 0.010 | 1.22 (1.07-1.39) | 0.003 |
| **EAC** |  |  |  |  |  |  |  |  |
| Married | Reference |  | Reference |  | Reference |  | Reference |  |
| Unmarried | 1.14 (1.07-1.22) | <0.001 | 1.18 (1.10-1.26) | <0.001 | 1.10 (1.02-1.18) | 0.011 | 1.13 (1.05-1.22) | 0.001 |
| **Others** |  |  |  |  |  |  |  |  |
| Married | Reference |  | Reference |  | Reference |  | Reference |  |
| Unmarried | 1.25 (1.00-1.55) | 0.048 | 1.36 (1.09-1.70) | 0.007 | 1.27 (1.01-1.60) | 0.041 | 1.39 (1.10-1.76) | 0.006 |
| **Grade** |  |  |  |  |  |  |  |  |
| **Well differentiated** | |  |  |  |  |  |  |  |
| Married | Reference |  | Reference |  | Reference |  | Reference |  |
| Unmarried | 1.13 (0.77-1.64) | 0.531 | 1.48 (0.99-2.23) | 0.057 | 1.18 (0.78-1.77) | 0.435 | 1.51 (0.97-2.35) | 0.070 |
| **Moderately differentiated** | |  |  |  |  |  |  |  |
| Married | Reference |  | Reference |  | Reference |  | Reference |  |
| Unmarried | 1.16 (1.06-1.27) | 0.001 | 1.18 (1.08-1.30) | <0.001 | 1.14 (1.03-1.25) | 0.010 | 1.15 (1.05-1.27) | 0.004 |
| **Poorly differentiated** | |  |  |  |  |  |  |  |
| Married | Reference |  | Reference |  | Reference |  | Reference |  |
| Unmarried | 1.17 (1.08-1.26) | <0.001 | 1.21 (1.12-1.31) | <0.001 | 1.13 (1.04-1.22) | <0.001 | 1.17 (1.08-1.26) | <0.001 |
| **Undifferentiated** |  |  |  |  |  |  |  |  |
| Married | Reference |  | Reference |  | Reference |  | Reference |  |
| Unmarried | 1.66 (0.72-3.83) | 0.236 | 1.64 (0.63-4.26) | 0.313 | 1.66 (0.72-3.83) | 0.236 | 1.64 (0.63-4.26) | 0.313 |
| **Location** |  |  |  |  |  |  |  |  |
| **Upper third of esophagus** | |  |  |  |  |  |  |  |
| Married | Reference |  | Reference |  | Reference |  | Reference |  |
| Unmarried | 1.06 (0.78-1.45) | 0.715 | 0.99 (0.72-1.36) | 0.944 | 1.11 (0.80-1.54) | 0.522 | 1.05 (0.75-1.46) | 0.796 |
| **Middle third of esophagus** | |  |  |  |  |  |  |  |
| Married | Reference |  | Reference |  | Reference |  | Reference |  |
| Unmarried | 1.15 (0.99-1.34) | 0.076 | 1.24 (1.06 -1.45) | 0.006 | 1.15 (0.98-1.35) | 0.096 | 1.24 (1.05-1.46) | 0.010 |
| **Lower third of esophagus** | |  |  |  |  |  |  |  |
| Married | Reference |  | Reference |  | Reference |  | Reference |  |
| Unmarried | 1.17 (1.09-1.24) | <0.001 | 1.20 (1.13-1.28) | <0.001 | 1.12 (1.05-1.20) | 0.001 | 1.16 (1.09-1.24) | <0.001 |
| **TNM Stage** |  |  |  |  |  |  |  |  |
| **Stage I** |  |  |  |  |  |  |  |  |
| Married | Reference |  | Reference |  | Reference |  | Reference |  |
| Unmarried | 1.23 (1.03-1.47) | 0.022 | 1.41 (1.18-1.69) | <0.001 | 1.22 (0.99-1.49) | 0.057 | 1.40 (1.14-1.72) | 0.001 |
| **Stage II** |  |  |  |  |  |  |  |  |
| Married | Reference |  | Reference |  | Reference |  | Reference |  |
| Unmarried | 1.16 (1.01-1.33) | 0.032 | 1.17 (1.02-1.35) | 0.026 | 1.14 (0.98-1.33) | 0.081 | 1.15 (0.99-1.34) | 0.064 |
| **Stage III** |  |  |  |  |  |  |  |  |
| Married | Reference |  | Reference |  | Reference |  | Reference |  |
| Unmarried | 1.22 (1.08-1.39) | 0.002 | 1.26 (1.11-1.43) | <0.001 | 1.16 (1.02-1.33) | 0.026 | 1.21 (1.06-1.38) | 0.006 |
| **Stage IV** |  |  |  |  |  |  |  |  |
| Married | Reference |  | Reference |  | Reference |  | Reference |  |
| Unmarried | 1.15 (1.06-1.24) | 0.001 | 1.15 (1.07-1.25) | <0.001 | 1.12 (1.04-1.22) | 0.005 | 1.13 (1.04-1.23) | 0.003 |
| **Therapy** |  |  |  |  |  |  |  |  |
| **Surgery and radiotherapy** | |  |  |  |  |  |  |  |
| Married | Reference |  | Reference |  | Reference |  | Reference |  |
| Unmarried | 1.07 (0.97-1.19) | 0.195 | 1.12 (1.01-1.25) | 0.029 | 1.16 (0.97-1.38) | 0.099 | 1.16 (0.97-1.38) | 0.099 |
| **Only surgery** |  |  |  |  |  |  |  |  |
| Married | Reference |  | Reference |  | Reference |  | Reference |  |
| Unmarried | 1.20 (1.06-1.36) | 0.004 | 1.15 (1.01-1.12) | 0.032 | 1.25 (0.94-1.65) | 0.125 | 1.23 (0.93-1.63) | 0.147 |
| **Only radiotherapy** | |  |  |  |  |  |  |  |
| Married | Reference |  | Reference |  | Reference |  | Reference |  |
| Unmarried | 1.11 (0.88-1.41) | 0.388 | 1.36 (1.05-1.34) | 0.021 | 2.09 (1.05-4.17) | 0.036 | 2.50 (1.15-5.43) | 0.021 |
| **No surgery or radiotherapy** | |  |  |  |  |  |  |  |
| Married | Reference |  | Reference |  | Reference |  | Reference |  |
| Unmarried | 1.14 (1.10-1.19) | <0.001 | 1.23 (1.18-1.29) | <0.001 | 1.16 (1.09-1.24) | <0.001 | 1.16 (1.08-1.24) | <0.001 |

OS=overall survival; CSS=cause-specific survival; ESCC=esophageal squamous cell carcinoma; EAC=esophageal adenocarcinoma; TNM= tumor, node and metastasis.
